# Evaluation of four clinical laboratory parameters for the diagnosis of myalgic encephalomyelitis

**DOI:** 10.1186/s12967-018-1696-z

**Published:** 2018-11-21

**Authors:** Kenny L. De Meirleir, Tatjana Mijatovic, Krishnamurthy Subramanian, Karen A. Schlauch, Vincent C. Lombardi

**Affiliations:** 1 Himmunitas Foundation, de Tyraslaan 111, 1120 Brussels, Belgium; 2 R.E.D. Laboratories, Z.1 Research park 100, 1731 Zellik, Belgium; 3 Himmunitas Clinic, de Tyraslaan 111, 1120 Brussels, Belgium; 4 Desert Research Institute, 2350 Raggio Pkwy, Reno, NV 89512 USA; 5 Department of Microbiology and Immunology, University of Nevada, Reno, School of Medicine, Reno, NV USA

**Keywords:** ME/CFS, Diagnostic, IL-8, PGE2, sCD14, CD57, Chronic fatigue

## Abstract

**Background:**

Myalgic encephalomyelitis (ME) is a complex and debilitating disease that often initially presents with flu-like symptoms, accompanied by incapacitating fatigue. Currently, there are no objective biomarkers or laboratory tests that can be used to unequivocally diagnosis ME; therefore, a diagnosis is made when a patient meets series of a costly and subjective inclusion and exclusion criteria. The purpose of the present study was to evaluate the utility of four clinical parameters in diagnosing ME.

**Methods:**

In the present study, we utilized logistic regression and classification and regression tree analysis to conduct a retrospective investigation of four clinical laboratory in 140 ME cases and 140 healthy controls.

**Results:**

Correlations between the covariates ranged between [− 0.26, 0.61]. The best model included the serum levels of the soluble form of CD14 (sCD14), serum levels of prostaglandin E2 (PGE_2_), and serum levels of interleukin 8, with coefficients 0.002, 0.249, and 0.005, respectively, and p-values of 3 × 10^−7^, 1 × 10^−5^, and 3 × 10^−3^, respectively.

**Conclusions:**

Our findings show that these parameters may help physicians in their diagnosis of ME and may additionally shed light on the pathophysiology of this disease.

**Electronic supplementary material:**

The online version of this article (10.1186/s12967-018-1696-z) contains supplementary material, which is available to authorized users.

## Background

Myalgic encephalomyelitis is a heterogeneous illness often characterized by a number of physical symptoms and comorbid conditions such as systemic inflammation, neurocognitive abnormalities, innate immune activation, and gastrointestinal issues [1]. Current estimates indicate that up to 4 million individuals in the U.S. are afflicted with ME [2], with an annual productivity loss in excess of $23 billion in the United States alone [3], underscoring the significant impact of ME as a major public health concern both economically and socially.

Little is known regarding the etiology of ME; however, a number of potential triggers or stressors are typically reported to coincide with the onset of the disease including viral or flu-like symptoms, chemical exposure, physical trauma, or emotional distress [4–6]. Based on genome-wide association studies (GWAS) and familial studies, a genetic predisposition has also been suggested to play a role in the disease [7–10]. With the exception of perhaps exercise intolerance and general inflammation, no ubiquitous physical symptoms or diagnostic biomarkers have been identified; therefore, a diagnosis is primarily based on meeting a number of inclusion and exclusion criteria [11, 12]. As such, ME is often considered a “heterogenous” disease and this limitation has hindered the identification of nonsubjective biomarkers [13]. Compounding this issue, current research suggests that previously described potential biomarkers, such as cytokines, change over time [14] and may vary depending on illness duration [15] and severity [16].

In light of the heterogenous nature of ME, a biomarker that separates patients into more homogenous subgroups, based on clinical presentation would be useful; albeit, some progress has been made in this area. For instance, Naviaux and coworkers conducted a metabolomics study of ME cases and controls and identified a profile, characterized by decreased circulating ceramides [17]. A later study by Nagy-Szakal et al. elaborated that the subset of ME cases who present with irritable bowel syndrome (IBS) comorbidity have increased plasma ceramides [18]. When taken together, these studies imply that serum or plasma ceramides may have the necessary sensitivity to subgroup ME cases based on gastrointestinal (GI) comorbidity, although these observations will need to be evaluated against diseases with overlapping symptomology to show specificity.

While not universally prevalent, a small number of immunological parameters have been frequently reported to associate with subgroups of ME cases including natural killer (NK) cell dysfunction and inflammatory cytokine production. For these reasons, as well as other, it is generally accepted that ME has immunological underpinnings [19–23]. Currently, the mechanisms responsible for these observations remain elusive, but if identified, this knowledge would provide a greater understanding of ME pathology, potentially leading to effective treatment options.

In the present study, we investigated four immunological parameters that were chosen based on the following rationale: Subjects with ME are often characterized by coinfections; therefore, we analyzed the chemotactic factor interlerukin-8 (IL-8), which is produced by macrophages and, to a lesser extent, by other cells in response to infection. In light of the GI comorbidity, commonly associated with ME we measured the soluble form of CD14 (sCD14); a surrogate marker of bacterial translocation in the gut [48]. Orthostatic intolerance and atypically cerebral vasoconstriction following orthostatic challenge are often observed in subjects with ME [24, 25]. Additionally, females commonly report exasperated symptoms of premenstrual syndrome and Th1 suppression is commonly observed as well, all of which are associated with prostaglandin E2 (PGE_2_) production [26–28]. Lastly, CD57 expression on natural killer (NK) cells is an end-stage marker for their maturation [29]. Previous studies suggest that circulating CD57^+^/CD3^−^ NK cells are increased in association with chronic infections but are downregulated in several autoimmune diseases [29]. Mounting evidence suggests that ME may have an “autoimmune-like” etiology [30–35]; therefore, we included circulating CD57^+^/CD3^−^ NK cells in our analysis.

## Methods

### Study design

Retrospective analyses of existing clinical data were conducted under a HIPAA authorization waiver, as determined by the University of Nevada, Reno Institutional Review Board (IRB) [Protocol 1213405-1]. From these data, four clinical parameters were chosen for investigation based on their potential diagnostic utility and their ability to subgroup ME cases, given their common symptoms and medical anamnesis. In the present study, the following data were collected: absolute counts of peripheral CD57^+^/CD3^−^ lymphocytes; serum levels of IL-8, (also known as CXCL8); serum levels of the soluble form of CD14 (sCD14) and serum levels of prostaglandin E2 (PGE_2_). Age and gender were also recorded to address potential age- and gender-related contributions.

### Clinical data

Whole blood (K_2_EDTA anticoagulant) and serum was collected by venipuncture from ME cases at the Himmunitas Foundation clinic (Brussels, Belgium), and transported the same day to R.E.D. Laboratories (Zellik, Belgium) for clinical analysis. These analyses were conducted as part of the patient’s initial clinical work-up. Laboratory results were search from a sequential accession series starting in 2013. From these data the first 70 female and 71 male cases who met the following inclusion criteria were selected: all four parameters were evaluated for each subject using blood drawn on the same day; and each subject had received a diagnosis of ME according the Canadian Consensus Criteria for ME [11]. Data from these 70 female cases (age median 44 years; age range 16–68 years) and 71 male cases (age median 43 years; age range 15–67 years) were used in the analysis, as well as data from 70 female (age median 44.5 years; age range 14–86 years) and 70 male (age median 43.5 years; age range 18–70 years) healthy subjects. Control subjects met the following inclusion criteria: Normal white blood cell count, no inflammation, (C-reactive protein < 1 mg/L), and no clinical history of chronic immune disease or diabetes.

### PGE_2_ quantitative determination in human serum

Serum levels of PGE_2_ were evaluated using the DetectX^®^ Prostaglandin E2 Immunoassay kit (Arbor Assay, Michigan, USA) according to the manufacturer’s instructions. The concentration of PGE_2_ in each diluted sample was calculated using a four-parameter logistic regression fitting routine provided with the microplate reader analysis software (BioRad, Nazareth, Belgium) and the neat concentrations of each sample were obtained by multiplying this value by the dilution factor. Normal values for the assay were previously determined by analyzing 79 de-identified samples from 42 female and 37 male healthy subjects referred to the laboratory for a general check-up screening. Statistical analyses established that no differences in PGE_2_ were linked to age; however, noticeable differences were linked to gender. Therefore, gender-specific median values were established. These median values were used to standardize all values in the format of a ratio. The control range was established using the 10th (P10) and 90th (P90) percentile of the healthy population as references values, i.e. P10 ratio to median as lower limit and the P90 ratio to median as upper limit (Table 1).

**Table 1:** Demographic information and clinical values for the respective study groups

	Age range	Age mean	CD57 (cells/mL)	sCD14 (ng/mL)	PGE_2_	IL-8 (pg/mL)
Reference range:			60–360	1430–2800	0.1–2.81* 0.17–6.45	0–15
Female controls (N = 70)	14–86	44.5	76	2654	1.83	13
Female cases (N = 69)	16–68	44	46	3425	7.97	1156
Male controls (N = 70)	18–70	43.5	103	2365	4.00	14
Male cases (N = 71)	15–67	43	58	2918	11.80	697

* PGE_2_ values are given as a ratio to a reference range median; ranges are for females (top) and males (bottom)

### Quantification of IL-8 and sCD14 serum levels

Serum levels of IL-8 were assessed as part of a multiplex cytokine panel using the BD Cytometric Bead Array Human Inflammatory Cytokines Kit on a BD FACS Canto II™ flow cytometer (Becton–Dickinson Biosciences, San José, CA, USA). Absolute values were calculated from a standard curve using FCAP Array™ software and inter-assay controls were used for each batch. Similarly, serum levels of sCD14 were assessed using BD Cytometric Bead Array Human Soluble CD14 Flex Set on the same cytometer, according to the manufacturer’s instructions. Positive controls and inter-assay controls were run for each batch, and unknown values were calculated from a standard curve generated using protein standards of known concentrations.

### Quantification of peripheral CD57^+^/CD3^−^ lymphocytes

Absolute CD57^+^/CD3^−^ lymphocytes counts were evaluated with a BD FACS Canto II™ flow cytometer on anticoagulated (K_2_EDTA) whole blood using a clinically validated lyse no wash protocol. Briefly, 50 µL of whole blood was added to BD Trucount™ absolute counting tubes, with 10 µL of PerCP-anti-CD45 (clone 2D1), 10 µL of PE-anti-CD3e (clone UCHT1) and 10 µL of FITC-anti-CD57 (clone HNK-1) antibody and incubated for 10 min in the dark. The samples where then and lysed with 0.5 mL of BD FACS™ lysing solution and analyzed promptly. Lymphocytes were gated based on forward and side scatter, collecting a minimum of 1000 counting beads, and the cells of interested were identified as the CD57^+^/CD3^−^ population. Counting beads in the Trucount™ tubes were used to calculate the absolute number of CD57^+^/CD3^−^ lymphocytes per ml of blood.

### Statistical methods

Our dataset consisted of the four variables measured for 70 female controls, 70 male controls, 70 female cases, and 71 male cases. One instance of the IL-8 measure (22,595,208 pg/mL) was 700-fold greater than the next highest IL-8 measure (32,717 pg/mL), and almost 12 standard deviations above the mean case IL-8 measure. This outlier was excluded from the dataset, leaving 69 female cases and 71 male cases. The data were preliminary analyzed using a logistic regression model. Additionally, in order to explore if a diagnostic algorithm could be established, we performed a classification analysis using the respective clinical variables in combination with age and gender as the covariates and subject status (case or control) as the target variable.

## Results

Distributions of the four variables were log-transformed and tested for normality. As none of the four distributions were normal, non-parametric statistical tests were performed. A simple forward and backward logistic regression was performed on the four biochemical parameters and the covariates age and gender. None of the four biochemical parameters were found to be in high correlation with each other, ranging between [− 0.26, 0.61]. The best model included the sCD14, PGE_2_, IL-8 measures with coefficients 0.002, 0.249, and 0.005, respectively, and p-values of 3 × 10^−7^, 1 × 10^−5^, and 3 × 10^−3^, respectively.

In order to establish if a potential diagnostic algorithm was feasible, we conducted a classification and regression tree (CART) analysis using sCD14, PGE_2_, IL-8 as predictive variables and subject status as the target variable. CART analysis utilizes recursive partitioning of these clinical variables into increasingly smaller sets of the dependent variable (case or control in our analysis). During each recursion, the binary splits for each dependent variable is examined and the split that optimizes the homogeneousness of the two resulting groups, with respect to the dependent variable is chosen [36]. CART analysis is well-suited for this investigation, as it produces an algorithm that is amenable for diagnostic data purposes. It is also able to classify systems that differ due to natural causes and determine the relative importance of different variables for identifying homogeneous groups within a data set [37].

**Fig. 1:**
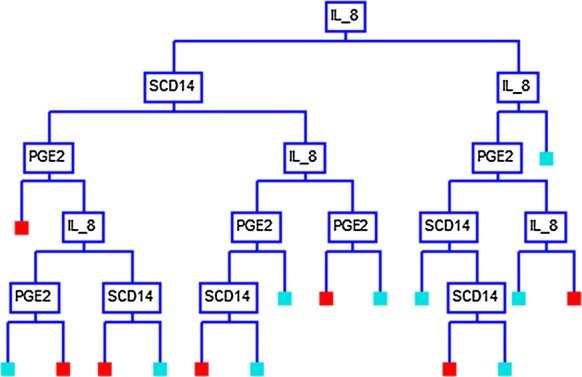
Decision tree produced using CART analysis. Each node represents a split value of the independent variable, which determines the optimal number cases or controls predicted by the analysis. Colored boxes represent the terminal point of the decision metric. Blue boxes represent cases and red boxes represent controls. A comprehensive version of the decision tree, which defines the predictive algorithm, is presented as Additional file 1: Figure S1

Using the CART analysis with the Gini splitting method, a 16-node decision tree produced a set of “if–then” logical (split) conditions, based on the biochemical parameters, that yielded approximately 90% specificity and sensitivity in distinguishing ME cases from controls (Table 2). Each node of the decision tree provided a decision metric based on the subject values of one of the three clinical parameters (Fig. 1). A detailed analysis of each node with the respective split values are given in Additional file 1: Figure S1.

**Table 2:** CART analysis summary of ME cases and healthy controls

Actual class	Total class	Percent correct (%)	Predicted classes	
			ME case	Control
			N = 139	N = 141
Case	140	89.29	125	15
Control	140	90.00	14	126
Total	280			
Average		89.64		
Overall correct		89.64		
Specificity		90.00		
Sensitivity		89.29		
Precision		89.93		
F1 statistic		89.61

We additionally conducted a CART analysis with all four clinical variables, as well as the demographic variables of age and gender; however, this analysis did not significantly improve the predictive outcome (data not shown).

## Discussion

Diagnosing ME is problematic in that no disease-specific biological markers have been described; therefore, a diagnosis is made when a patient meets a combination of costly and subjective inclusion and exclusion criteria [11, 12, 38]. For this reason, a convenient diagnostic for ME would dramatically improve patient care and decrease medical costs. Such a diagnostic would also allow physicians and researchers to objectively follow the efficacy of new therapeutics during the course of clinical trials. With this in mind, we have conducted a retrospective analysis to assess the utility of four clinical laboratory parameters in diagnosing ME cases. Three of these were ultimately used to conduct a CART analysis in order to identify an algorithm that can assist physicians in the use of these parameters.

When we conducted our analysis, access to similar data from other disease cohorts was not available. These data would be necessary to show that the parameters can be used to distinguish ME from other diseases with overlapping comorbidities, and thus, represented the principal limitation to our study. Additionally, because this was a retrospective study that utilized existing data, we were not able to establish subgroups based on clinical presentations. The only data available were clinical laboratory data, demographic data and disease diagnosis. Although acquiring and screening specimens representing these other disease cohorts will be the focus of future studies, the data and results presented here are an important first step in the ultimate goal of developing a specific diagnostic for ME. This study also provides clear unequivocal evidence of the immunological underpinnings of ME.

In this study, we analyzed data from 140 ME cases and 140 healthy controls subjects, composed of approximately half males and half females in each group. Using the two analytical methods, linear regression and CART, we determined that gender and age did not significantly impact the predictive model, nor did the inclusion of absolute CD57^+^/CD3^−^ lymphocytes counts. The CD57 antigen is a sulfated glycan carbohydrate epitope and among circulating lymphocytes, is primarily expressed on mature NK cells and terminally differentiated T cells. Although its function on NK cells is still a subject of ongoing research, CD57 expression is also considered a marker for NK cells that are highly mature and terminally differentiated [39], and is also generally believed to reflect their immunosenescence [40]. NK cell dysfunction has been reported to associated with ME by several investigators [41, 42]; however, recent studies have brought into question their diagnostic potential [43]. While differences in CD57^+^/CD3^−^ lymphocytes counts have been associated with other neuroimmune diseases [44], their inclusion in our study did not significantly improve its overall diagnostic ability. Nevertheless, there may be a number of reasons for this. In the present study, we limited our subjects to those with a confirmed diagnosis of ME, which by definition, implies that other potential causes have been ruled out [11]. Notwithstanding, when investigating diseases with overlapping or similar symptoms, including CD57^+^/CD3^−^ lymphocyte counts may be beneficial. For instance, Blom et al. reported that tick-borne encephalitis virus-induced NK cell activation is primarily limited to differentiated CD57^+^/CD56^dim+^ NK cells [45]. Additionally, CD57^+^ NK cells are routinely implicated in herpesvirus infections [46, 47]. Therefore, including this parameter may be of utility when establishing or ruling out an initial diagnosis of ME.

In this study, we utilized classification and regression tree CART analysis to produce a predictive binary decision tree, that defines the optimal values of each parameter as a branch point. Using serum IL-8 as the first node in the decision tree and with a cut-off value of > 137.5 pg/mL, 109 of the 140 ME cases subjects were correctly categorized. Moreover, 132 of 140 controls were correctly categorized as having IL-8 values of ≤ 137.5 pg/mL (Additional file 1: Figure S1).

IL-8 is a chemotactic factor that attracts leukocytes, such as neutrophils, basophils, and T-cells to sites of tissue injury and infection. Upregulation of IL-8 has also been reported in association with several chronic diseases such as autoimmune and inflammatory disease [48]. Multiple isoforms of IL-8 are produced through alternate splicing and proteolytic cleavage; a process that is largely cell-specific. For instance, the most common isoform in humans, IL-8(6–77), is produced primarily by monocytes and lymphocytes, while IL-8(1–77), which is produced by fibroblasts and endothelial cells, is likely the second most abundant form [49]. Notwithstanding, a wide variety of other cells express IL-8 including neutrophils, fibroblasts, mast cells, smooth muscle cells and dendritic cells [50–54]. Additionally, posttranslational modification of IL-8, such as citrullination or deamination, is known to lead to altered biological activity [55]. Understanding which isoform of IL-8, and which posttranslational modifications are most prevalent in ME may provide valuable insight into the underlying pathophysiology of the disease.

In our retrospective analysis, we observed IL-8 to be significantly upregulated in a large number of ME subjects; however, previous studies have reported inconsistent or contradictory results with respect to IL-8 [19, 23, 56–58]. For instance, in one study, no difference in serum IL-8 levels were observed in ME cases when compared to healthy controls [16]; however, another study using the same analytical methods, showed significant differences between short-duration versus long-duration ME cases versus controls [15]. These discrepancies may be the result of patient selection methods, choice of sample matrix (i.e. serum vs. plasma, vs. mRNA) or analytical methods. Indeed, the heterogenous nature of the disease makes patient selection a perennial difficulty when investigating ME and the two aforementioned studies support this assertion, at least with respect to IL-8.

In that two previously mentioned studies utilized the same multiplex product (custom Luminex 51-plex manufactured by Affymetrix), and their mean ME results were significantly lower than that observed in our study, we speculated that the discrepancy may be inherent in assay systems. Bead-based and sandwich ELISAs rely on antibodies pairs that are specific for two different epitopes on the target protein, therefore, it is reasonable to conclude that some antibody pairs may not detect all isoforms of a protein. To explore this possibility, we purchased an Affymetrix ProcartaPlex™ Simplex IL-8 Luminex kit, which utilizes the same bead (region 27) as that used in the multiplex kit. We then screened the serum of 15 ME cases that were part of the present study and also included two recombinant IL-8 isoforms as standards; IL-8(6–77) and IL-8(1–77). Both isoforms were detected by the assay; albeit, IL-8(1–77) was observed to be approximately 40% of that reported by the manufacturer (1956 pg/mL observed vs 5000 pg/mL expected). We also screened the 15 ME cases using a commercial ELISA kit (Invitrogen, IL-8 Human ELISA Kit) and observed the 15 ME cases to yield different results between the three assay methods, with the results produced by the Affymetrix kit being significantly lower than the other two methods, consistent with the discrepancy between our study and two previously mentioned studies (Additional file 2: Table S1). While we did not resolve the issue of potential IL-8 isotype differences, it does suggest that the observed differences between the three studies are likely the result of a combination of factors, including patient selection and analytical methods. It also emphasizes the reliance of the methods described herein when implementing the predictive model as well as suggests further investigations with respect to IL-8 are warranted in ME.

In additional to CD57^+^/CD3^−^ lymphocytes and serum IL-8, we also investigated the utility of including sCD14 and PGE_2_ in our model. CD14, along with Toll-like receptor (TLR)-4 and lymphocyte antigen 96 (MD-2), forms the receptor complex that bind lipopolysaccharides (LPS) [59], which are found in the outer membrane of Gram-negative bacteria. Previous reports have identified sCD14 as a nonspecific marker of monocyte activation [60] as well as a surrogate marker of bacterial translocation in the gut [61]. Therefore, individuals with ME and who present with an upregulation of sCD14 are likely to have a GI comorbidity. Accordingly, sCD14 may be a useful biomarker for subgrouping ME cases, with irritable bowel syndrome. PGE_2_, on the other hand, regulates multiple aspects of inflammation and the functions of different immune cells [62]. It is generally acknowledged as a mediator of acute inflammation, stimulating chemotaxis and activation of mast cells, neutrophils, and macrophages, during the early stages of inflammation [63–65]. It also has the capacity promote the induction of suppressive IL-10 as well as the downregulation of several proinflammatory cytokines, thus suppressing nonspecific inflammation [62]. Although it can promote the activation, maturation and migration of professional antigen-presenting cells, it has been shown to suppress both innate and antigen-specific immunity at several levels [62, 66]. Therefore, PGE_2_ has proinflammatory as well as immunosuppressive properties.

Our model showed that sCD14 can further subgroup ME cases with IL-8 levels that satisfy the following inequality: 22.0 pg/mL < IL-8 ≤ 137.5 pg/mL. We also observed that PGE_2_ can be used to subgroup ME cases with IL-8 levels that satisfy the following inequality: 137.5 pg/mL < IL-8 ≤ 240.0 pg/mL (Additional file 1: Figure S1). Additional combinations of IL-8, sCD14 and PGE_2_, as articulated in Additional file 1: Figure S1, can be used to further subgroup ME cases from healthy controls.

## Conclusions

In summary, we have conducted a retrospective analysis of four clinical parameters from ME cases and healthy controls and have shown that three of these parameters can be used to delineate ME cases and controls with approximately 90% specificity and sensitivity using the analytical methods described herein. Further studies will be necessary to show if the proposed model will be useful in diagnosing ME from diseases with overlapping comorbidity.

## Additional files

**Additional file 1: Figure S1.** Comprehensive version of the decision tree presented in Fig. 1.

**Additional file 2: Table S1.** Comparison between three analytical methods. All values are pg/mL.

## Abbreviations

CART: classification and regression tree
CXCL8: chemokine (C–X–C motif) ligand 8
GWAS: genome-wide association studies
HIPAA: Health Insurance Portability and Accountability Act
IL: interleukin
IRB: Institutional Review Board
LPS: lipopolysaccharides
ME: myalgic encephalomyelitis
PGE_2_: prostaglandin E2
sCD14: soluble cluster of differentiation 14
TLR: toll-like receptor
