# Supplementary figures and images for: Evaluation of four clinical laboratory parameters for the diagnosis of myalgic encephalomyelitis
Source: J Transl Med. 2018 Nov 21;16:322. doi: 10.1186/s12967-018-1696-z (PMC6249861; doi:10.1186/s12967-018-1696-z)

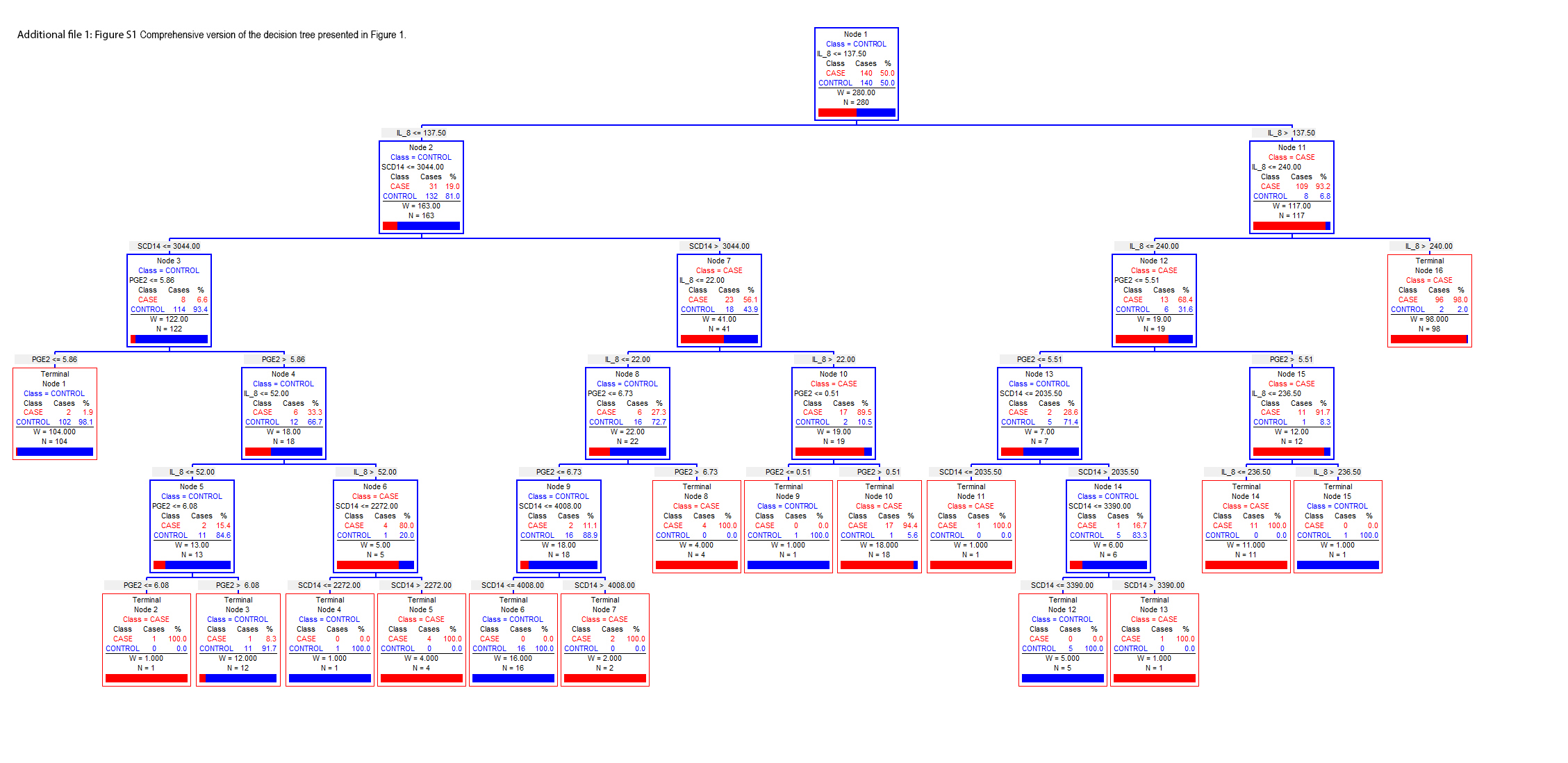

Supplement: Supplementary file 1 — Additional file 1: Figure S1. Comprehensive version of the decision tree presented in Fig. 1. [file 12967_2018_1696_MOESM1_ESM.jpg]
