# Supplementary material for: Evaluation of four clinical laboratory parameters for the diagnosis of myalgic encephalomyelitis
Source: J Transl Med. 2018 Nov 21;16:322. doi: 10.1186/s12967-018-1696-z (PMC6249861; doi:10.1186/s12967-018-1696-z)
Supplement: Supplementary file 2 — Additional file 2: Table S1. Comparison between three analytical methods. All values are pg/mL. [file 12967_2018_1696_MOESM2_ESM.docx]

| **Additional file 2: Table S1.** Comparison between three analytical methods. All values are pg/mL. | | | |
| --- | --- | --- | --- |
| **Sample number** | **IL-8 ELISA**  **Invitrogen** | **IL-8 Flow**  **BD** | **IL-8 Luminex** |
| ME-1 | 9.4 | 10.9 | 16.1 |
| ME-2 | 6.5 | 8.1 | 1.3 |
| ME-3 | 739 | 1292 | 16.1 |
| ME-4 | 8.3 | 14.7 | 1.3 |
| ME-5 | 916 | 978 | 10.1 |
| ME-6 | 4880 | 508 | 30 |
| ME-7 | 1668 | 1657 | 29.9 |
| ME-8 | 1391 | 3096 | 16.8 |
| ME-9 | 1886 | 2885 | 23.5 |
| ME-10 | 1144 | 1596 | 43.9 |
| ME-11 | 7.9 | 15 | 1.3 |
| ME-12 | 4.1 | 9 | 1.3 |
| ME-13 | 1539 | 1777 | 17.3 |
| ME-14 | 1201 | 1292 | 17.2 |
| ME-15 | 883 | 1925 | 13.9 |
| Mean | 1085 | 1137 | 16 |
